# Headspace for parents: qualitative report investigating the use of a mindfulness-based app for managing parents’ stress during COVID-19

**DOI:** 10.1192/bjo.2021.1070

**Published:** 2021-12-17

**Authors:** Abigail Burgess, Kate Cavanagh, Clara Strauss, Bonamy R. Oliver

**Affiliations:** UCL Institute of Education, University College London, UK; School of Psychology, University of Sussex, UK; UCL Institute of Education, University College London, UK

**Keywords:** Adjustment disorders, childhood experience, conduct disorders, psychosocial interventions, qualitative research

## Abstract

**Background:**

Stress can compromise parental well-being and may contribute to harsh and critical parenting styles, which are in turn associated with children's conduct problems. Coronavirus disease 2019 (COVID-19)-related restrictions are likely to have exacerbated parental stress as, for many, UK-based family life was altered considerably. Mindfulness has been demonstrated to improve stress management and emotion regulation when delivered to parents in person, however, more accessible online interventions are under-researched.

**Aims:**

To provide preliminary data on family well-being and parent–child relationships as well as the acceptability and usability of the Headspace app – a self-delivered mindfulness-based intervention – for parents in low-risk families during the early days of the COVID-19 pandemic.

**Method:**

We provided 12 parents with access to Headspace, and collected qualitative data (semi-structured interviews and 5 minute speech samples) immediately following the initial COVID-19 lockdown in the UK. The resulting transcripts were thematically analysed.

**Results:**

Most parents reported Headspace to be acceptable and useful – improvements in parents’ own sleep were particularly noted – and there was high adherence to the intervention. However, difficulties related to family well-being and parent–child relationships following the lockdown were also reported.

**Conclusions:**

As a result of the confounding impact of COVID-19 restrictions, and varied access to app content, we were unable to determine any outcomes to be a result of practising mindfulness specifically. However, COVID-19 has had a profound impact on many UK-based families, including those previously at low risk, and our results demonstrate that Headspace may have beneficial effects for parents. There is a need to more rigorously test this tool with a broader range of families.

## Background

Worldwide, Coronavirus disease 2019 (COVID-19) and associated lockdown restrictions are projected to have serious long-term implications for the mental health and well-being of a range of families and young children, including those who may not have been classified as vulnerable prior to the pandemic (‘low risk’).^1^ We report results from a small study with parents of young children, originally designed as a case series to understand the experiences, feasibility and acceptability of the Headspace app (hereon, ‘Headspace’) – a self-delivered, mindfulness-based intervention (MBI). We present a qualitative report, as planned quantitative data collection was patchy, unreliable and uninterpretable in this small sample at this tumultuous time, but full qualitative data was achieved through one-to-one interviews. We aim to provide unique insight into the use of Headspace, as well as family well-being and parent–child relationships for these low-risk families over the period during and immediately after the first UK COVID-19 lockdown.

## Parental stress and mindfulness

Already an existing crisis, the mental health of children and young people is of grave concern in the post-pandemic era,^2^ with both internalising problems (such as depression, anxiety) and externalising (behavioural) problems seen as common concerns.^2^ Parent-focused parenting strategies (for example harshness and criticism) are associated with both increased internalising and externalising problems in neurotypical children.^3^ Parental stress can exacerbate the use of these negative parenting strategies,^4^ making it a key target for intervention, arguably in particular as parental stress has been heightened by COVID-19.^5^

Mindfulness is characterised by non-judgemental awareness and acceptance of present-moment experiences.^6^ MBIs aim to cultivate mindfulness through meditation practices and teacher-led discussion, with a wealth of evidence showing beneficial effects on stress, mental health and well-being, including self-help mindfulness resources, in non-clinical^7,8^ and clinical settings.^9^ The skills taught in MBIs have promise in the parenting context as, in addition to beneficial effects on stress, it is proposed that mindfulness improves emotion regulation, and self-efficacy^10^ – furthering their potential to interrupt negative parent–child cycles.^11^

## Mode of delivery

When delivered to parents, MBIs are most often delivered in group format, in person, and to families at risk for serious mental health difficulties, or to those with neurodiverse children who are thought to experience higher levels of parenting stress.^12^ This is not surprising given that most MBIs are based on the clinical mindfulness-based stress reduction framework, which involves a high level of facilitator expertise.^13^ However, self-directed MBIs are gaining ground, particularly in non-clinical settings, offering the potential for efficient approaches to intervention and prevention.^7^ Demonstrating the efficacy of preventative interventions is acknowledged to be difficult,^14^ but prevention is an increasingly key priority in mental healthcare and research in the UK,^15^ and we argue may be of critical importance in the parenting setting for children's well-being. Yet, despite low-risk families not being entirely impervious to mental health challenges^16^ – arguably particularly following pandemic-enforced social restrictions – there is a lack of research into the effects of online, self-delivered MBIs for parents of neurotypically developing children, in low-risk families.^12^ We aim to bridge this research gap.

Online, self-delivered, interventions are proposed to be both more accessible and cost-effective than in-person approaches,^17^ yet there are concerns in non-clinical populations around effectiveness and poor engagement.^18^ Regarding mindfulness specifically, concerns are that practice without therapist guidance may result in a greater frequency of adverse events,^19^ although this may be mitigated by using guided meditations.^20^ We propose that increasing the acceptability of remote, self-directed interventions may lie in addressing preconceptions of digital tools, as hypothetical acceptability is often lower than actual acceptability on their implementation.^21^ As these forms of intervention are relatively novel, there is a need for wider research to ascertain the best way to implement them in practice.^17^

## The current study

**Fig. 1:**
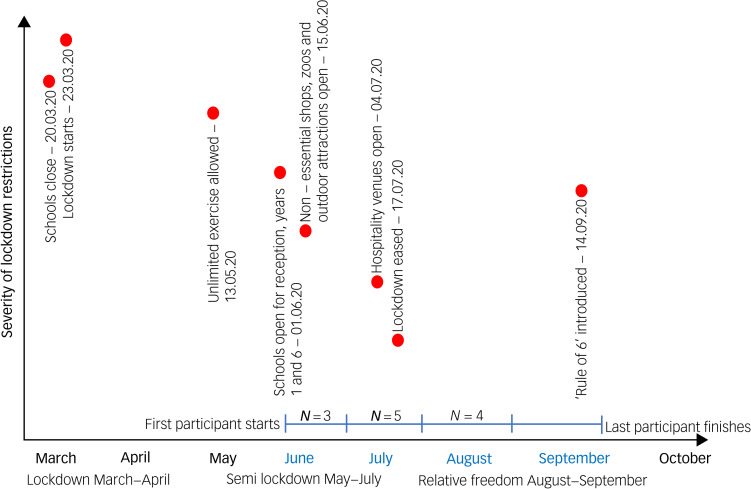
Visualisation representing lockdown restrictions in the UK over the course of March 2020 to October 2020 and the impact on data collection. This visualisation of the different stages of lockdown restrictions in the UK in 2020 was created by the authors of this paper using the dates of family-specific significant restrictions as recorded by the Health Foundation's COVID-19 Policy Tracker (2021). The ‘Rule of 6’ refers to a measure introduced by the UK Government intended to control the spread of COVID-19, meaning that apart from a limited number of exceptions (including, for example, work and education), any social gatherings of more than six people were against the law while the ‘rule of 6’ was in force. This was enforceable by the police, who were given powers to disperse such gatherings and fine attendees.

Headspace provides self-directed content designed to support mindfulness practice alongside a broader suite of materials supporting mental well-being and performance (such as exercise and sleep) and is the best-evidenced self-help app available to the general population.^22^ In randomised controlled trials, Headspace has been demonstrated in diverse adult populations to produce small between-group post-intervention differences in favour of Headspace compared with control groups for stress (*g* = 0.24), anxiety (*g* = 0.21) and depression symptoms (*g* = 0.36).^23^ Although its effectiveness has been tested in a wide variety of clinical and non-clinical contexts,^22^ despite its relevance and promise, nothing has yet been conducted with parents. Thus, we aimed to investigate the use of Headspace in a small number of low-risk families, collecting in-depth, qualitative data to ascertain parents’ experiences of Headspace, and of family well-being and parent–child relationships, in order to answer the research question, what are parents of young children's experiences using a self-directed mindfulness app to manage their stress, in relation to their experiences of being a parent? The timing of data collection – during the UK's initial response to COVID-19 – additionally provided an insight into family life over the period when the first UK lockdown was beginning to be eased, and immediately after it had been lifted. Lockdown restrictions and data-collection points are visualised in Fig. 1, to contextualise the discussion of our results.

## Method

### Design

This study examined the use of Headspace by a small number of families in the UK, collecting in-depth, qualitative data and app usage data to ascertain its acceptability and usability. Written, informed consent was obtained from all participants.

Thematic analysis was employed as this method is not tied to one epistemological or ontological position, and is useful in identifying overarching themes across participant responses.^24^ Coding was conducted in six stages^24^ by the first author who also facilitated the data collection, utilising an iterative and inductive approach to the data to produce a rich and encompassing reflection of participants experiences. Collaborative confirmation of the final coding was made by the first and last authors. However, independent second coding was not conducted as we took a social constructionist approach to analysis, acknowledging the role of the researcher in the interview, and the perspectival nature of the findings.^24,25^

### Ethics statement

The authors assert that all procedures contributing to this work comply with the ethical standards of the relevant national and institutional committees on human experimentation and with the Helsinki Declaration of 1975, as revised in 2008. All procedures involving human patients were approved by the Department of Psychology at Goldsmiths, University of London (Ethics Committee Approval: PS080120ABS).

### Participants

Via social media, we recruited a volunteer sample of UK-based parents (aged over 18) of children aged 2–5 years old, with no self-reported, pre-existing, neurodevelopmental or mental health conditions, with access to an internet-enabled smart device, and no experience of meditation or use of Headspace in the past 6 months. A pre-determined sample size of 12 was achieved as this is proposed to be sufficient to incorporate into a thematic analysis,^26^ and simultaneously small enough for the originally planned case series.^27^

A demographically homogeneous sample of 12 parents (from 12 families) were recruited, 11 of whom identified as White or White British, were married or cohabiting with a partner, and were mothers (one single parent and one father). Mean parental age was 42 years (s.d. = 3.58 years) and mean target child age was 3.5 years (s.d. = 1.08 years). Six (50%) participants reported being single-child households, three had a second child under 1 year old, and three had a second child older than 5 years. Most (*n* = 11) parents reported being educated to at least undergraduate degree level (half to either master (*n* = 4) or doctoral degree levels (*n* = 2)), and all parents were employed at the time of the study. Participants were given free access to Headspace for the duration of the study and were further incentivised on completion with a voucher worth £15.

### Materials

The 5 minute speech samples (FMSS)^28^ were collected from parents pre and post the Headspace intervention. Participants were telephoned or video-called and asked to talk for 5 minutes uninterrupted, answering the following prompt: ʻPlease tell me what you've been thinking and feeling about (your child) in the past two weeks. What your relationship with (your child) has been like, and how well you have gotten along with them in the last two weeks'. Participants also engaged in a semi-structured interview at a 2-week follow-up telephone or video call, lasting for between 30 and 45 minutes. The topic guide is available in the Supplementary materials.

Audio-recordings for the pre- and post-intervention FMSS and semi-structured interviews were transcribed and anonymised before being coded. As a result of the aforementioned impact of the fluctuations in social restrictions because of COVID-19 on the reliability and interpretability of our planned data, we did not quantitatively score these transcripts for pre-/post-differences. Instead, here, the FMSS transcripts were analysed in conjunction with the semi-structured interview transcripts to provide a rich insight into family well-being and the parent–child relationship in our small, low-risk sample at this tumultuous time in the UK.

### Intervention

Headspace contains a wide variety of meditative and non-meditative content, including guided meditations, sleep aids, music/sounds and short animations, as well as some content designed for parents and their children to do together. Participants were asked to complete the ʻBasics' packages 1, 2 and 3 as a guided introduction to mindfulness. Each of these packages lasts for 10 days, thus the intervention period was 30 days. In the Basics package 1, participants were able to choose between 3, 5, or 10 minutes of meditation a day, and in Basics 2 and 3, from 10 or 15 minutes a day. No specification was given for the duration of the mediation chosen, and no limitations were placed on participants’ use of the rest of the app's content.

## Results

**Fig. 2:**
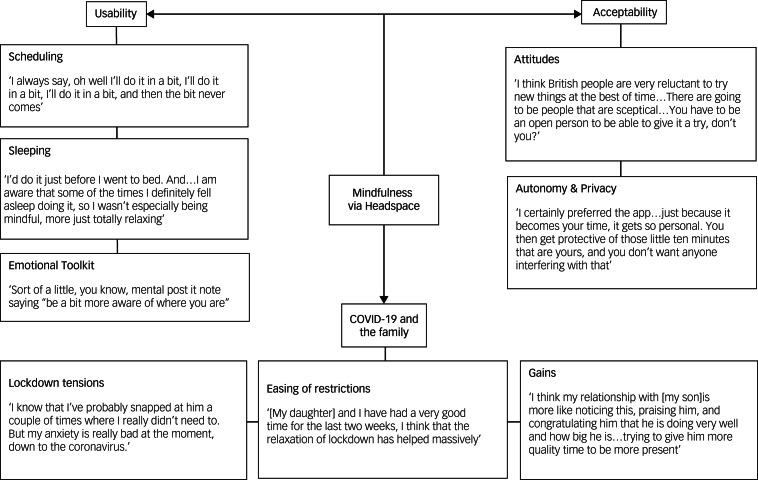
Thematic map depicting the most widely discussed key themes and subthemes representing parents’ experiences with illustrative quotes (see Supplementary Table 1 for more detail).

Supplementary Table 1 available at https://doi.org/10.1192/bjo.2021.1070 depicts the full results of the thematic analysis of both the FMSS and the semi-structured interview data. Fig. 2 visualises a thematic map of the key themes identified.

### Acceptability and usability

Participants reported Headspace as acceptable and broadly feasible to do, a finding also evidenced by the high number of ʻBasics' sessions participants completed out of the 30 they were requested to do (Mean 26 Basics sessions, s.d. = 11.84, ranging from 1 to 43 sessions). Six participants also used the sleep content (completing an average of 4 sessions, s.d. = 9.01, although the range was also large, from 1 to 29 sessions). Three participants also used the app with their children, although this was less frequent (completing an average of one children's meditation session, s.d. = 1.08, ranging from one to three sessions), with two participants also using the children's sleep content specifically (completing an average of one session, s.d. = 1.27, ranging from one to four sessions).

There were just two reports of negative experiences using Headspace, but these are important to note as they are not often recorded in the extant literature.^19^ One participant reported associating physical sensations with breathing difficulties linked to asthma:

ʻConcentrating on my breath … I was getting … that sort of feeling of relaxation and sort of slightly … ugh it's not quite the right word, but almost … sort of … giddiness, I guess? … I would find myself worrying that I was getting that feeling not because I was meditating but because I was breathing incorrectly or something. Which I know is a strange thing that ties in with that feeling of asthma, and that feeling of, if I'm concentrating on my breathing, there's something hardwired in my brain to be saying to me, what's wrong? … Are you ok? Are you breathing enough?' (P12)

Of note, there is some move away from using the breath as the only anchor point in mindfulness teaching,^29^ and our results suggest that this may indeed be pertinent for some people. The other negative experience was centred around sleep disturbance. Although the participant did not report this as lasting, it is noteworthy as they found it to be distressing and negatively impactful on their perception of mindfulness:

ʻI was doing it last thing at night for quite a while, and I wasn't sleeping afterwards. It was waking me up, rather than calming me down … my husband noticed it and he was like, “you've got to stop doing them at night” … when I stopped doing that at night, it was noticeable that my sleep went back to being fine.' (P2)

This was markedly different from the majority of participants (*n* = 11) who reported the app to be beneficial for their sleep. Although noting a feeling that the meditation was ‘gearing them up’ for the day ahead, most parents (*n* = 9) nonetheless found it easiest to schedule into their night-time routine. Irrespective of the time of day, parents reported getting a better quality of sleep as a result of practising mindfulness, and, although not the intention, those using the app at night specifically spoke of being lulled into sleep more quickly as a result – and commonly of falling asleep during the exercise:

ʻI think if I'd been able to do that daily practice at the beginning of the day … it would have been even more helpful because, although … it was probably really helpful in terms of getting to sleep,  … I think … my challenge was hanging on to it for the next day, and staying awake!' (P5)

ʻI definitely felt more patient, actually. I think it, I don't know whether it just helps me sleep … a lot quicker, and very soundly.' (P1)

Although our qualitative data suggests that parents experienced a reduction in stress over the course of this study, as a result of fluctuating government restrictions in the UK (see Fig. 1), it was not possible to determine whether these findings were a result of using Headspace, or a result of the timing of data collection. However, most parents (*n* = 8) attributed the reduction in their stress to be a result of learning to be mindful during an emotionally complex lockdown, including perceiving vicarious utility for their children's emotion management skills:

ʻ … if I'm feeling fear, I can do this for me, if I'm feeling anxious, I can do this, I can focus, if I can't sleep, I've got these tools. That's how it feels to me, it's like a whole load of tools, I've got some extra tools in my belt now, as a parent, that I can use to make me a more rational parent, a calmer parent, a more focused parent, and [we are] starting to get some of those tools into my daughter's bag that's going to help her regulate her emotions and communicate clearly what she needs and what she feels. So, I think I'm very grateful for the experience that we had.' (P4)

Further to most parents reporting the intervention as helpful, its method of delivery – i.e. self-directed via an app – was also evaluated positively (*n* = 11), with some participants explicitly describing it as preferable to participating in person because of the privacy and autonomy, as well as the convenience, of being able to access the intervention on their phone:

ʻ … it was quite nice to be able to do it without anyone there that could potentially judge, I guess. Nobody had to know I was doing it, whereas, if you go to a group, they do … ' (P2)

ʻI think it's actually quite a vulnerable thing. Like just sitting there in silence, like, you know, it only takes one person to cough, or sniff, or, and … I think I'd step out of where I was quite quickly. But, with this, you know, you're in your own world which I really like.' (P1)

### COVID-19 and the family

We found Headspace to be widely reported as both feasible in parents’ daily life and acceptable (*n* = 11). However, some parents (*n* = 6) explicitly stated that the specific – and unforeseeable – timing of our study in terms of COVID-19 had an impact on their experiences. Some (*n* = 2) explained that they would have found it more useful to have tried this in advance of the lockdown:

ʻI think it's quite an interesting study for this time. I think if it had've been a year earlier, it would have been very useful for COVID!' (P2)

Others (*n* = 4) found that, in fact, access to Headspace was timely:

ʻ … it's unfortunate your study's coming now, but in terms of the lockdown, and like people being isolated and alone … there's some amazing content on there.' (P1)

We were also able to gain some insight into the impact of COVID-19 specifically in terms of participants’ relationships with their children. Strikingly, these experiences can be delineated into the different stages of the lockdown during which they participated – immediately following the full UK lockdown, followed by the relaxation of rules in the summer and then the transition for children back into school and nursery (see Fig. 1 for the numbers of participants recruited at each stage).

### Semi-lockdown: June and July 2020

Semi-lockdown was largely considered a period of significant uncertainty. Family tensions directly attributed by parents to the effects of the pandemic were most often (*n* = 7) reported as difficulties caused by the various and fluctuating Governmental rules during this period:

ʻIn all honesty, the past two weeks have been a little bit tricky … I think the current situation we're in in terms of a semi-lockdown could be part of the problem. [My son] … seems to be struggling a little bit … maybe a little bit more frustrated at times.' (P5)

ʻ … we'll see some of his friends, but we can't see other friends … he's finding that harder to understand, and therefore there's been more sulking and tantrums going on. Because sometimes we can go, and sometimes it's a no, and I can see how that's harder to understand.' (P7)

For some (*n* = 4), these tensions were gendered, in that mothers reported feeling the burden of responsibility fell to them rather than their partners, as intense pressure and guilt to be both a mother and balance work, as well as household administration:

ʻI'm just at the computer and I'm on the phone to people, which is horribly dull, and I always feel really bad. But at the moment, I can't help it – we are just juggling things … I'm often the one that's not doing the fun things … I have to be the practical one, I have to be the one working, I have to be the one juggling things.' (P5)

### School summer holiday: August 2020

Over the summer holiday, some family pressures were relieved by being able to take time off during a period when restrictions were significantly reduced. The most notable reported change was on the quality of children's sleep and their behaviour (*n* = 4):

ʻ … we've been on [summer] holiday … So we took her to the park, and we took her to the zoo, we went out and played in the local playground in the place where we went to go and stay and she … just absolutely adored every second. And I think that it's just so lovely to see her light up and wake up every morning and have had, you know, 12, 13 h of sleep and 3 h naps a day just because she's been so wiped out from having so much fun.' (P1)

In addition, the role that access to extra-familial support played in improving their children's behaviour was widely reported (*n* = 11):

ʻWe had a visit from his Aunt yesterday, and, you know, he was just delighted, and the change to his behaviour was quite stark.' (P4)

ʻ … now we're sort of gone through lockdown, my mum has sort of been looking after [my daughter] and that has a massive impact on my relationship and how we are together, like how I view her, because I've got time away as well. And you can have that little break and come back to it and you're like oh my gosh I've missed you so much! These are all the things I love about you!' (P3)

### Return to school: September 2020

Some parents (*n* = 7) reported finding their children's return to nursery or school to be challenging – especially following the extended absences earlier in the year, and the continued difficulties in accessing regular child care:

ʻ … we've been doing a fair amount of preparing for school, there's been a bit of a balancing act that we haven't had for a very long time because of COVID, of my husband starting back at work in his school, so therefore having to work out child care with our usual methods not being quite as accessible as they were before. So, that's been a little bit of a struggle …' (P2)

The consequences of school returns were experienced by some (*n* = 3) as difficulties with sleep once again:

ʻI think, you know, coming alongside going to nursery and being so stimulated [after lockdown ended], her sleep hasn't been amazing which is really unlike her. So, we've had a handful of really bad nights which, honestly, was absolutely draining and exhausting.' (P1)

### Relative freedom: August to September 2020

Importantly, however, there were also reports of positive experiences during the pandemic, notably on participants’ perceptions of their children, and their attitude to life more broadly. For example, some mothers (*n* = 6) noticed and appreciated the sensitivity of their child to their – the mother's – distress, and the resultant empathy after what has been a difficult year at home:

ʻ … he saw me crying because I … had enough, I was too overwhelmed with work, and he comes to me and says – he gives me a cuddle, and says – ʻMummy, I'm here'. Like the same way that I do when he is upset … and it was so sweet. He is a good boy.' (P8)

ʻHe has been really good since I was poorly last week, and he likes to pretend to be a doctor. So, he'll come along with his toys and he'll go, “open your mouth”, and check me out, and he's really caring – finishes it off with a sticker for being a good girl, and off he goes again.' (P11)

Appreciating the ʻlittle things' was also reported by parents (*n* = 7) as a change to the way they perceived their family that they may not have experienced without the enforced lockdown:

ʻI think COVID's really helped in the kind of simple pleasures and the simple joys of just appreciating on a child's level maybe, which is often quite tricky in a busy life. COVID's given us a chance to calm down a bit and have a reset.' (P2)

ʻWe've had a couple of little ʻsleep overs' [with each other, during lockdown], which is her favourite activity at the moment … So, she'll only go to bed about an hour later than she normally would, but it's so special for her because there's so few things we can do, so that's really nice.' (P2)

## Discussion

In a low-risk – i.e., well-educated, socioeconomically advantaged – UK sample, we explored the use of a self-directed meditation app, Headspace. We provide preliminary, qualitative evidence that these parents found Headspace to be both feasible and acceptable. Parents also perceived a positive impact of learning mindfulness on their emotion regulation skills and stress tolerance. We discuss our results in terms of the utility of Headspace, but arguably of greatest interest is that the nature and timing of data collection, although having a negative impact on our study plans, provided key insights into life for low-risk families at an unsettled time in the UK because of COVID-19. These novel insights are important for understanding the impact of COVID-19 and associated restrictions on families who may not have been classified as vulnerable pre-pandemic^1^. Our results are discussed in the context of assertions that, for socioeconomically disadvantaged children and their parents, the risk of adverse consequences are likely far greater.^30^

### Headspace for families

We found encouraging parent perceptions of effectiveness, and adherence to, Headspace, although we note these are very preliminary findings in need of replication with robust study designs and larger, more diverse samples. Importantly, caution in generalising is advisable, not only in light of our privileged and self-selecting participants, but also as they had multiple contacts with researchers that may have positively influenced intervention effects and app adherence.^23^ Nonetheless, our findings offer some support for previous assertions that online MBIs can have beneficial effects on stress,^31^ as well as for the suggestion that hypothetical acceptability of digital interventions may be lower than actual acceptability.^21^ We speculate our findings also hint at the potential utility of remote MBIs in the post-pandemic era, although again we remain mindful that the nature of our sample provided the requisite cultural and financial capital supporting greater adherence to self-directed interventions.^32^ As such, we emphasise the importance of robust testing of Headspace with a more diverse sample of parents to better understand its effects in a more pragmatic context.

### Mechanisms of action

One of the mechanisms by which mindfulness is thought to improve family functioning is via non-judgemental attention of the self and child, such attention fostering an awareness of the distinction between perception and affective response.^11^ It is therefore interesting that, following Headspace, parents in our sample specifically spoke about appreciating ʻthe little things', and their perceptions of the value this perspective added to interactions with their child. This is supported by previous research suggesting a dispositional capacity to maintain a focus on the present is associated with reductions in parental coercion and improvements in parental warmth.^10^ This present-moment focus may also go further and be indirectly related to improvements in parenting and family functioning by reducing impulsivity, improving marital and co-parenting relationships and depressive symptoms.^33^

It was, however, difficult to tease apart the effects of the Headspace sleep content from the mindfulness content in our sample as half the participants used both, and many participants reported incidentally finding the mindfulness content to be a sleep aid. This was reported both as parents perceiving experiences of better quality of sleep owing to improvements in emotion regulation after practising mindfulness, and as an incidental aid to sleep whereby parents fell asleep during the exercise as they found it very soothing. If replicated, this may be of importance since evidence suggests practising mindfulness is itself associated with improved sleep and decreased sleep-interfering cognitive processes such as rumination.^34^ However, this evidence is heterogeneous and does not clearly demonstrate direct effects on sleep quality and duration.^34^ As such, more research is required to investigate the mediators and moderators of the effects we anticipate following a mindfulness intervention for parents of young children.^35^ Specifically, the inclusion of measures of mindfulness will help to better understand the mechanism by which parents experience improvement in their sleep.

### COVID-19 and ‘low-risk’ family life

Despite the fact that our small sample was demographically homogeneous and socioeconomically advantaged (for example none of the parents reported economic loss ensuing from the restrictions), our qualitative findings revealed parent perceptions of a substantial negative impact of COVID-19 and associated measures on their families’ well-being. The reported experiences of increased parenting stress in these families is striking when considered in their low-risk context, as the effects of the pandemic on family life are likely to be highly influenced by socioeconomic circumstance: families less economically and socially ʻwell placed' may be expected to have been faring worse.^36^

Contrarily, it is possible that more advantaged parents experience perceptions of difficulties – albeit from different sources than those less economically advantaged – that nevertheless have an impact for these families. For example, research with parents from high-income countries with higher educational attainment has demonstrated that they spent more time parenting their children than other parents, arguably a function of both more flexible jobs, and societal pressure.^37^ These pressures may have been directly exacerbated as a result of the pandemic-induced shift to home working and home schooling and may therefore be a unique risk factor for families of higher socioeconomic status. We also propose that pandemic-related difficulties faced by low-risk families may be more transitory than those at higher-risk, because of the greater personal, financial and social resources available to them.^38^

Although health inequalities following pandemics are most notable for people living in poverty as opposed to privilege,^39^ the far-reaching effects of COVID-19 restrictions on family life are apparent even in these low-risk families. In particular, challenges were often reported related to sleep difficulties (of the parent or child). Importantly for stress management, previous findings suggest a lower quality of parental sleep is associated with higher levels of stress, as well as less observed positive parenting.^40^ We therefore theorise that Headspace-induced improvements in sleep (as well as mindfulness, as discussed above) may also have the potential to reduce parental stress and increase positive parenting, in turn potentially improving child adjustment outcomes.

### Limitations

Although we offer evidence that the use of Headspace to manage parental stress was both acceptable and feasible for parents of young children in our sample, we acknowledge the limitations of this study. In addition to the aforementioned effects of data-collection timing and the low risk, privileged nature of our sample, all of which limit generalisability, our participants used the app in a variety of different ways, accessing content in addition to the basic mindfulness packages they were directed to use. It was therefore difficult to ascertain what parts of the app, if any, were responsible for reported stress reductions. Moreover, although qualitative work is an essential step in evaluating the effectiveness of a novel intervention,^27^ the limitations of our design, in conjunction with COVID-19-induced study limitations, are acknowledged. As such, although we posit the theoretical promise of Headspace for reducing parental stress and in turn improving children's outcomes, the current study was not designed to test this, and we emphasise that robust and diverse research in this area is needed. We aim and hope for the current study to be a springboard for more rigorous, well-powered, randomised controlled studies.

### Implications

COVID-19 is likely to have a long-lasting and widespread impact on the availability of preventative physical and mental healthcare while health services recover from emergency-oriented care.^41^ Previously, mindfulness has been demonstrated to be beneficial for both parent and child outcomes, via reductions in stress and improvements in emotion regulation.^15,16^ Here, we provide preliminary support for the acceptability, feasibility and utility of an online, self-directed MBI for parents experiencing stress. We suggest it should be a priority to build on the current findings in diverse families, but particularly for families who were vulnerable before the pandemic, and are now significantly more at risk.^2^ In addition, better understanding of the relationship between sleep and mindfulness in family dynamics and well-being will be key. In so doing, we hope that mindfulness-based apps, such as Headspace, may prove to be accessible, inexpensive and potentially useful self-directed interventions for parental stress and ultimately child well-being and mental health for diverse families.

## Data Availability

The data that support the findings of this study are available on reasonable request and with appropriate confidentiality restrictions from the corresponding author, A.B. The data are not publicly available owing to their containing information that could compromise the privacy of research participants.

## Supplementary material

For supplementary material accompanying this paper visit https://doi.org/10.1192/bjo.2021.1070. click here to view supplementary material
