# Supplementary material for: Headspace for parents: qualitative report investigating the use of a mindfulness-based app for managing parents’ stress during COVID-19
Source: BJPsych Open. 2021 Dec 17;8(1):e15. doi: 10.1192/bjo.2021.1070 (PMC8692845; doi:10.1192/bjo.2021.1070)
Supplement: Supplementary file 1 [file bjosup.zip › S205647242101070Xsup001.pdf]

Semi-structured Interview Topic Guide – Feasibility and acceptability of integrating Headspace into daily life

1. How did you find using the Headspace app?

   How feasible was it to use on a daily basis?

   What barriers did you face to completing the sessions daily?

   What facilitated you to complete the sessions?

2. How did you find doing the basics?

   What was helpful about it?

   What was difficult about it?

   What would have made it easier for you to do?

3. What was your experience of learning and practicing mindfulness skills?

   What was positive about incorporating mindfulness practice into daily life?

   Where there any negative aspects to incorporating mindfulness practice into daily life?

   Was there a specific time/place you found it easier to do mindfulness at/in?

   Did you explore the app? If so, was there a specific pack you found most helpful? Why?

4. Do you think that doing Mindfulness could benefit other parents of young children?

   Why?

   In what way?

   If not, why not?

5. Do you think that using a smartphone app to deliver mindfulness sessions is helpful?

   Why?

   In what way?

   If not, why not?

6. Do you have any other advice for us about how we can improve parents' experience of using the Headspace app?
7. Do you have any other advice for us about how we can improve parents' experience of participating in this study in general?

TABLE S1

Table expanding on the themes and subthemes identified from a thematic analysis of participants semi-structured interviews and five-minute speech samples

**Theme**	**Sub-Theme**	**Brief Description**	**N (12)**	**Illustrative Quote**
Acceptability	Attitudes	Being open minded and a belief in *Headspace* helps; framing is paramount.	11	"I think British people are very reluctant to try new things at the best of times…There are going to be people that are sceptical, and from the get go are going to go, this isn't going to work for me, and then it won't work for them. You have to be an open person to be able to give it a try, don't you?"
		Derogatory perceptions of mindfulness, trends in the public consciousness, often related to age.	5	"…when I told my dad I was doing this, he was like, meditating?! Why are you doing that?! And it was just the way he asked me, it was almost like I'd said, oh, you know, I take drugs twice a week or something, you know"<br><br>"It's like veganism, isn't it? It's all that kind of…I don't know, hippy sounds like a derogatory word for it, but you know what I mean, just slightly more that way. But yeah, I think it's just become a bit more fashionable…"
	Negative reactions	Asthma-related and sleep disturbance	2	"…because of…my asthma…Concentrating on my breath, I found, threw up a lot of thoughts, certainly at first. I should say, because of the breathing thing—and this happened less towards the end, though it was still happening occasionally—I must have met a resistance—I don't think I was—but I was getting a…getting that sort of feeling of relaxation and sort of slightly…ugh it's not quite the right word, but almost…sort of…giddiness, I guess? I'm not sure if that's quite right. And certainly at first, while I was in the process, I would find myself worrying that I was getting that feeling not because I was meditating but because I was breathing incorrectly or something. Which I know is a strange thing that ties in with that (laughs) feeling of asthma, and that feeling of, if I'm concentrating on my breathing, there's something hardwired in my brain to be saying to me, what's wrong? You know? Are you ok? Are you breathing enough?"

				"I was doing it last thing at night for quite a while, and I wasn't sleeping afterwards. It was waking me up, rather than calming me down…I don't really know why they were keeping me awake, it didn't seem logical, because I think the actual process was actually quite calming….I mean, my husband noticed it and he was like, you've got to stop doing them at night. I don't know why, it doesn't make sense, but, the thing that should be calming was keeping me awake. But it was noticeably that, so when I stopped doing that at night, it was noticeable that my sleep went back to being fine."
Usability	Autonomy & Privacy	Parents appreciated the autonomy and privacy of using an app to learn mindfulness in their own space	11	"[I] found the actual class itself [in-person] quite…intimidating is not the right word, but…I found myself quite self-conscious of it. So, it was quite nice to be able to do it without anyone there that could potentially judge, I guess."
	Scheduling	Demands on parents' time are great; recognition of these struggles by *Headspace* may help	12	"Yeah! I always say, oh well I'll do it in a bit, I'll do it in a bit, I'll do it in a bit, and then the bit never comes. There's always something else that I have to put first, whether it's my son, or cooking, or cleaning, there's always something like oh I really should be doing that, I shouldn't sit down, I need to be prepping this, doing that. And it's difficult to put myself first, it really is…"<br><br>"I felt guilty in some moments in the beginning that I struggled to start, because I'm like, oh my god…I'm just tired and…it's so easy to go to do it for a few minutes but I can't stop with three minutes to lay down, and do it properly. Maybe something that accepts that being a mother and doing work and being with kids is difficult. It's difficult to change behaviours and the way that you fit things that you know that are healthy and that can help you on a daily basis…yeah. Change is really difficult!"
	Routine	Incentive and embedding *Headspace* in routine increased likelihood of adherence.	10	"…it is a short amount of time, but I know I would have gone, oh I'm just really tired, or oh I'm just not in the mood for it, or something, so, you know, because it's contributing to something else, it was easier to stay more disciplined."<br><br>"I think the impetus of this study that you're doing, and having committed to it for someone else was really useful. And I think, for me to do it, and I think the results of that have been really useful to push

				me to want to continue it"
	Sleeping	Fitting *Headspace* in at night was incidentally beneficial for sleep.	10	"I definitely felt more patient, actually. I think it, I don't know whether it just helps me sleep—fall asleep, it doesn't necessarily keep me asleep—but it certainly helped me fall asleep a lot quicker, and very soundly, which definitely helped when my daughter was waking me up in the middle of the night for two hours. And I didn't feel stressed or anything, I just felt very calm about the fact that, oh well, I'm going to be up now and, you know, just got to parent in the middle of the night. Whereas before, I would have been irritated, I could have easily been irritated by the fact that my sleep had been disturbed. And I have like mild sleep anxiety, so…I think it really helped with that. And just rationalising a lot of it."<br><br>"I'd always do it once the boys had gone to bed, so I only ever did it in the evening. Because…it wouldn't be possible to do it at any other time. And quite often, I'd do it just before I went to bed. And which, I have to say, I am aware that some of the times I definitely fell asleep doing it, so I wasn't especially being mindful, more just totally relaxing. But, that also sort of has benefits because sometimes it takes quite a long time before I go to sleep, so then knowing that it definitely helped me get off to sleep, I then had to concentrate on, I was like, this isn't to put you to sleep, this is supposed to be concentrating on, you know, calming yourself down, not just actually falling asleep—that's kind of cheating."
	Emotional Toolkit	*Headspace* improved emotion regulation and stress management skills	11	"So, I feel, I feel maybe like I'm a better parent, in the sense that I am a calmer parent. I can notice things better with him. I was always very proud of him, but I'm even more proud."<br><br>"Sort of a little, you know, mental post it note saying "be a bit more aware of where you are", and especially at—it tended to be after work, when maybe my wife was making dinner for the kids and I had a half an hour or something to play with them and ask them how their day was and that sort of thing. It tended to be there more, so if I was giving him a cuddle on the sofa watching CBeebies or whatever, it'd almost be then that the mental post it note would kick in and that feeling of actually being present when you're being cuddled by and cuddling people that you sort of love unreservedly, it's

				quite a powerful feeling. And just that mental note to do that and, you know, be present at these times when, you know, your brain is programmed to give you all these endorphins and all you have to do is sit there and enjoy them! That was really powerful.”
	As Exercise	Parallels between mindfulness as a mental exercise, and physical exercise; effort increases benefits	10	“…it’s…like doing my physical exercise. It may not be the most appealing thing to start with, when you’ve got other pressures on your time, but once you’ve done it, you feel better and you start to see the benefits in other parts of your life as well.”<br><br>“But from that moment when you actually manage a little bit, and then you manage a little bit more, and then a little bit more, it compensates for the initial frustration of, I can’t do this, and I need to do something else. And then it’s like running, the more you run, the longer you want to run. So it was challenging at the beginning because I couldn’t really disconnect, but now that I can connect, I want to see how long can I be focussed.”
	Reciprocal benefits	Noticeable benefits for their child/ren—even where no children’s content was accessed.	9	“It helps me, and actually, it helps him. Now, sometimes, he says, when I know that he’s getting frustrated and I say, do you have to do something? And he says, yes, I have to count to ten. And he counts to ten, and he gets a bit calmer. Sometimes, he even imitates me when I’m like [breathes deeply] and he goes [childish deep breath]. [Spoken to the child] “You’re learning!”. It’s skills that are getting passed from me to him.”<br><br>“We’ve also been spending time together meditating…because of the app, we’ve found that there is a monster meditation that we can enjoy together. So, she’s really been learning those skills about calming herself down, about taking a deep breathe, about belly breathing, and things like that. We’re doing the app together as well, which I think has really helped. Like it’s helped her manage her emotions, which helps me feel better about it because she’s got a little bit more control, and that gives both of us a bit more patience with each other. So, things like that where I can see her relaxing as well, makes me feel so happy that she’s developing those skills and I think that helps our relationship massively.”

	Me time	Refocusing onto themselves, sometimes as parents this was a difficult adjustment	10	““One thing that I found really helpful was the time to focus on myself. So, regardless of whether it’s then a change in terms of approach, or in terms of, I suppose my own thought process maybe, and the fact that I don’t leave the house at all at the moment still, to have just a little bit of time to focus on me was actually really quite nice.”<br><br>“At least when my partner had my son, I was going, right, my son is fine, don’t need to worry about him for these ten minutes. I can put him right to the back. No need to worry about him. When it’s just me and him, I’ve still got that mom-dar—mom-radar—going on.”
	Self-evaluation	Negative evaluations of their capacity to be mindful; feelings of selfishness and failure	9	“Initially, the fact that it challenges you to just be present in the moment, and not think about, and not let your mind wander, I found that quite challenging. And it felt like you were failing all the time.”<br><br>“Well, yeah, because it’s for self, isn’t it? The whole point of it is that it’s for self, and that’s not something that I'm attuned to. We’ve always done stuff as a family. And I know that it’s meant to interlink, but we’ve always done stuff either for the family, or as a family. So to suddenly be like [imitates snooty voice], well actually, I need to go and do this…I dunno, it didn’t quite…”
	Timing	Challenges inherent with the pandemic; prevention better than cure	6	“Yeah, it’s an interesting one as a kind of, I think it’s quite an interesting study for this time. I think if it’d had’ve been a year earlier, it would have been very useful for COVID!”<br><br>“Like, timing wise, with my daughter going to school, there couldn’t be a better time for me to try something to relax, to stay calm, to not be caught up in my mind so much. Like, literally, for me, this is the biggest challenge I've come to face yet, sending her to school, so there’s been other things that I know other people would find difficult, but for me, this is the time that I've found most difficult, so having something that, like, helps me, because like at night time, that is when I'd be thinking the most, ruminating, like I would lie there for hours just with my own thoughts, running away with it.”
Lockdown Tensions	Juggling act	Complication of home life with addition of home	11	“…according to him [my son] my job’s very boring! Because I’m just at the computer and I’m on the phone to people, which is horribly dull, and I always feel really bad. But at the moment, I can’t help it—we are just juggling things. It’s not a normal time. And I think that makes it very difficult for everybody.

		working and schooling—particularly for mothers		So, I'm often the one that's not doing the fun things. When his dad's home, he's home and he's not working. And so he gets to be the fun one! Whereas I have to be the practical one, I have to be the one working, I have to be the one juggling things, or dropping him off at nursery and walking away for the day. I'm that one. And I think there are times when I feel that probably more than him, that I have to be the boring one, I have to be the practical one, I have to be the one that makes sure he gets washed and has to go to bed and think about what he's going to eat, and that he's watched too much TV."<br><br>"I feel like I need to be more on it as a mum, in terms of being able to support and help him through"
	Acting out	Increases in externalising behaviours and sleep problems for children	11	"…he does something that I totally hate, which is whining. Oh my God. Since he returned to school, after the lockdown, it has been horrendous! If I say no to something, he starts whining. And I say, "don't whine, if you whine, I'm not going to listen to you", so he whines even more, and I'm like, "stop". I can't deal with this! I can't deal with all this whining! And so today I have discovered when talking to the teacher in the nursery that she was like, "Yeah, since they've come back, there's loads of people whining".
		Confusing rules increased children's frustration with mixed messages	7	"The past two weeks. In all honesty, the past two weeks have been a little bit tricky. I'm not 100% sure why. I think the current situation we're in in terms of a semi-lockdown could be part of the problem. But [my son] I think seems to be struggling a little bit. And what I mean by that is he is less able to control his…maybe a little bit more frustrated at times."<br><br>"…we'll see some of his friends but we can't see other friends and I think he's finding that harder to understand and therefore there's been more sulking and tantrums going on because sometimes we can go and sometimes it's a no and I can see how that's harder to understand."
	Missing childcare	Childcare increased pressures on parents; anxieties around delays for social development, and	11	"…his attitude towards them [extended relatives] is different, so he won't accept being disciplined by them. He doesn't particularly want much attention from them. He'll still choose me over them, whereas he used to be quite happy to go off with any of my family and leave me behind and not worry

		the importance of visiting relatives		about it. So, I think that we, my son and I, have got closer during lockdown, but almost at the detriment of his relationships with everybody else which is a shame. But, I hope it'll get a bit better."<br><br>"It's—now we're sort of gone through lockdown, my mum has sort of been looking after her and that has a massive impact on my relationship and how we are together, like how I view her, because I've got time away as well. And you can have that little break and come back to it and you're like oh my gosh I've missed you so much! These are all the things I love about you! And it's just so nice to get"
Gains	It's the little things	Simple pleasures become important when there are few pleasures; pressure eased by home working	7	"We've enjoyed baking. Just being together as a family. I think COVID's really helped in the kind of simple pleasures and the simple joys of just appreciating on a child's level maybe, which is often quite tricky in a busy life—COVID's given us a chance to calm down a bit and have a reset."<br><br>"We've had a couple of little sleep overs [with each other, during lockdown], which is her favourite activity at the moment, and really cute…So, she'll only go to bed about an hour later than she normally would, but it's so special for her because there's so few things we can do that's really nice."
	Empathy	Empathy of children to increased stress and anxiety noted	6	"…the other day when he saw me, he saw me crying because I at some point I had enough, I was too overwhelmed with work, and he comes to me and says—he gives me a cuddle and says—"Mummy, I'm here". Like the same way that I do when he is upset, and I'll go "I'm here, I'm here. It's not ok, but it will be ok", and he kind of did the same actually, and it was so sweet. He is a good boy."<br><br>"He has been really good since I was poorly last week, and he likes to pretend to be a doctor. So he'll come along with his toys and he'll go, "open your mouth" and check me out, and he's really caring—finishes it off with a sticker for being a good girl, and off he goes again."
Easing of restrictions	Transition	Transition from lockdown to "normal life" difficult, especially in relation to childcare.	7	"I think that perhaps we're all a little bit apprehensive of the school start, and things are about to change again after like a long period where we've just all been together. And, you know, I think in the last couple of days I've realised how nice it's been. But it's also been really hard."

		Children's resilience and adaptability notable.	11	"He's just been coping really well, and he's not going to go back to nursery before school, and he's coped really well with that."
	Developing in Lockdown	Children developed immensely over lockdown—noticeably on recontact with relatives	10	"I noticed him as a bigger boy. We travelled for a holiday and then we met my parents and—that we didn't see in the last year—so I could notice a lot of difference in his behaviour and relationships with other adults. I'm very glad because he seems more polite, he seems more patient with things, he seems more careful with his Grandpa. In that same time, I felt a bit like the time is passing by quickly—that's always a little bit emotional."
